# Suppression of feline coronavirus replication in vitro by cyclosporin A

**DOI:** 10.1186/1297-9716-43-41

**Published:** 2012-04-30

**Authors:** Yoshikazu Tanaka, Yuka Sato, Shuichi Osawa, Mai Inoue, Satoka Tanaka, Takashi Sasaki

**Affiliations:** 1 Department of Veterinary Hygiene, Veterinary School, Nippon Veterinary and Life Science University, 1-7-1 Kyounan, Musashino, Tokyo, 180-8602, Japan; 2 Department of Infection Control Science, Faculty of Medicine, Juntendo University, Tokyo, 113-8421, Japan

## Abstract

The feline infectious peritonitis virus (FIPV) is a member of the feline coronavirus family that causes FIP, which is incurable and fatal in cats. Cyclosporin A (CsA), an immunosuppressive agent that targets the nuclear factor pathway of activated T-cells (NF-AT) to bind cellular cyclophilins (CyP), dose-dependently inhibited FIPV replication in vitro. FK506 (an immunosuppressor of the pathway that binds cellular FK506-binding protein (FKBP) but not CyP) did not affect FIPV replication. Neither cell growth nor viability changed in the presence of either CsA or FK506, and these factors did not affect the NF-AT pathway in fcwf-4 cells. Therefore, CsA does not seem to exert inhibitory effects via the NF-AT pathway. In conclusion, CsA inhibited FIPV replication in vitro and further studies are needed to verify the practical value of CsA as an anti-FIPV treatment in vivo.

## Introduction

Coronaviruses are single-stranded RNA viruses that generally cause respiratory or intestinal infections such as severe acute respiratory syndrome (SARS) in humans and transmissible gastroenteritis (TGE) in pigs. Feline coronaviruses (FCoV) have been classified into two biotypes, comprising the ubiquitous feline enteric coronavirus (FECV) and infectious peritonitis virus (FIPV). The widely accepted theory in vitro is that FIPV arises by mutation of parental FECV in the gastrointestinal tract of infected cats, then systemically spreads and causes FIP that is fatal in cats [1,2]. Although the mutation sites are not fully understood, some accessory genes are candidates for the site of the critical mutations responsible for FIP [3,4]. Approximately 80 % of all purebred cats are infected with feline coronavirus and among these, 5-12 % develop the classical symptoms of effusive/wet FIP, the non-effusive/dry form of FIP, or a combination of the two [5].

Many strategies have been developed to cure FIP. Interferon ω inhibits FIPV in vitro but is ineffective in vivo [6]. Various other immunosuppressants, such as glucocorticoids and cyclophosphamide, have been studied, but although these drugs prolong life, the outcome of FIPV infection remains fatal [7]. Thus, an effective vaccine and therapeutic medicine against FIPV are still needed.

Investigators have reported that the immunosuppressant cyclosporin A (CsA) can suppress the genomic replication and transcription of several viruses [8-11]. Cyclophilins (CyP) are cellular factors with high affinity for CsA [12] and comprise a family of cellular peptidyl-prolyl *cis-trans* isomerases (PPIases) that catalyze the *cis-trans* interconversion of the amino-terminal of peptide bonds to proline residues, thus facilitating changes in protein conformation as a chaperone protein [13]. Cyclosporin A blocks both the enzymatic activities of CyP that lead to the calcineurin (CN)-NF-AT and P-glycoprotein pathways. The capsid protein of human immunodeficiency virus type 1 (HIV-1) possesses a cyclophilin A (CyPA) binding site for incorporation into the virion [14,15]. A CsA-induced reduction in CyPA inhibits transport of the reverse-transcribed viral genome to the nucleus [16]. Cyclophilin B (CyPB) is another target of CsA that promotes hepatitis C virus (HCV) replication by regulating the RNA-binding ability of the HCV NS5B protein. In addition, CyPA facilitates mouse cytomegalovirus (MCMV) replication and CyPB is required for the infectious entry of human papillomavirus type 16.

Here, we show that CsA inhibits intracellular replication of the FIPV genome and viral protein expression in vitro independently of the NF-AT pathway.

## Materials and methods

### Cell culture and virus

*Felis catus* whole fetus-4 (fcwf-4; American Type Culture Collection, VA, USA) cells were maintained in Dulbecco’s modified Eagle’s medium (D-MEM, Sigma-Aldrich, Tokyo, Japan) supplemented with 10 % fetal bovine serum (JRH, Nissui, Tokyo, Japan). We propagated FIPV (79‐1146 strain; a gift from Dr Tsutomu Hodatsu, Kitasato University, Japan) in fcwf-4 cells and then purified them by linear sucrose gradient ultracentrifugation.

### Cells treated with cyclosporin A, cyclosporin H, and FK506

We inoculated fcwf-4 cells with FIPV 79–1146 at a multiplicity of infection (MOI) of 1 plaque-forming unit (pfu) per cell to study their effects on FIPV infection. After adsorption for 1 h at 37°C, the medium containing the virus was removed, and the cells were rinsed three times with phosphate-buffered saline [PBS (−)] and incubated with or without various concentrations of CsA (Sigma-Aldrich), cyclosporin H (CsH; Cosmobio, Tokyo, Japan) and FK506 (Sigma-Aldrich) for 20 h. The cells were then processed for photography. The cells were adsorbed with FIPV, rinsed three times with PBS (−), overlaid with DMEM containing 5 % fetal bovine serum and 2 % Agarose S (Nippon Gene, Toyama, Japan). After a 20-h incubation, the cells were stained with Giemsa solution and the plaques were counted.

### Plasmid constructs

We isolated the nucleocapsid (N) gene of FIPV from the plasmid pBRDI1 (provided by Dr Peter J. M. Rottier, Institute of Biomembranes, Utrecht University, The Netherlands) containing the FIPV 79–1146 genome, using the polymerase chain reaction (PCR) with the primers 5’-ACAAGGACGACGACGACAAGGCCACACAGGGACAACGCG-3’ and 5’-CCGGAATTCTTAGTTCGTAACCTCATCAA-3’ for the first amplification. The products of the first amplification were purified by electrophoresis and gel extraction, and then a second PCR proceeded with the purified products and primers containing a FLAG-tagged sequence: (5’-GCCACCATGGACTACAAGGACGACGACGACAAG-3’ and 5’-CCGGAATTCTTAGTTCGTAACCTCATCAA-3’). The second PCR products were cut with Nco I and Eco RI and subcloned into the sites between Nco I and Eco RI of the pTri-EX 1.1 vector (Novagen, Takara Bio, Kyoto, Japan) to express FLAG-tagged N protein.

### Real-time, quantitative reverse transcription-PCR (qRT-PCR)

The fcwf-4 cells were infected at an MOI of 1 pfu per cell and then incubated with or without CsA, CsH or FK506. The medium was removed at 22 h post-infection, and RNAiso-plus (Takara Bio) was added to the cells for RNA preparation according to the manufacturer’s protocol. Total RNA (800 ng) was reverse-transcribed using the PrimeScript RT-PCR kit (Perfect Real Time; Takara Bio). Viral cDNA were quantified by real-time PCR using the forward and reverse primers to the FIPV-N gene (5’-TGGCCACACAGGGACAAC-3’) and (5’-AGAACGACCACGTCTTTTGGAA-3’), and the TaqMan probe (FAM-GTTGCA GCACAGCCAGCATAAACAA-BHQ-1). Reaction mixtures were prepared according to the manufacturer’s protocol using Premix EXTaq (Takara Bio), and sequences were amplified using a 7500 Sequence Detection System (Applied Biosystems, Tokyo, Japan) under the following cycling conditions: initial denaturation at 95°C for 10 s and 40 cycles at 95°C for 5 s and 60°C for 36 s each. Complementary DNA to the FIPV-N gene was cloned into the pTri-EX 1.1 vector, which was serially diluted to provide standards for FIPV gene quantification. The viral RNA copy number was normalized using the feline β-2-microglobulin (β2M) gene (GenBank; NM_001009876). The β2M gene derived from fcwf-4 cells was cloned by PCR amplification using the following primers: fβ2M-F 5’-GGCGCGTTTTGTGGTCTTGGTC-3’ and fβ2M-R 5’-CACTTAACGACCTTGGGCTC-3’. The amplified PCR products were subcloned into pTAC-1 plasmids (BioDynamics Laboratory Inc. Tokyo, Japan) to provide standards for the β2M gene. We then quantified the feline β2M gene by real-time PCR using the forward (5’-CGCGTTTTGTGGTCTTGGTCTTGGT-3’) and reverse (5’-AAACCTGAACCTTTGGAGAATGC-3’) primers for the β2M gene and detected the gene using the TaqMan probe, TAMRA-CGGACTGCTCTATCTGTCCCACCTGGA-BHQ-2.

### Luciferase assay

Luciferase activities were quantified using pGL4.30 [luc2P/NFAT-RE/Hygro] (Promega, Tokyo, Japan), pRL-SV40 vectors for the NF-AT response assay and the interferon stimulation response was determined using the plasmid pISRE-TK hRluc (F) provided by RIKEN BioResource Center (Tsukuba, Japan) and the pGL3 promoter (Promega). Both reporter assays proceeded using the Dual-Luciferase Reporter Assay System (Promega). Briefly, the two reporter plasmids were co-transfected into fcwf-4 cells with or without CsA or FK506 for each assay. Recombinant feline IFNα (PBL Biomedical Laboratories, NJ, USA) was added at a concentration of 10 units/mL to the culture medium to evaluate the response to interferon (IFN). Total cell lysates were prepared with Reporter Lysis buffer provided with the Dual-Luciferase Reporter Assay System at 48 h before the assay. Luciferase activities were quantified in triplicate assays using a Lumat LB9507 (Berthold Technologies, Tokyo, Japan).

### Western blotting

The cell membranes were disrupted with cell lysis buffer [10 mM Tris–HCl, pH 7.8, 1 mM ethylenediamine tetraacetic acid (EDTA), 1 % NP-40, 0.15 M NaCl] including Complete Mini (Roche Diagnostics, Tokyo, Japan) at 20 h after infection. The cell lysates were resolved by electrophoresis on 4-12 % Bis-Tris gels (Invitrogen, CA, USA) and Western blotted onto Immobilon-P membranes (Millipore, Tokyo, Japan). Non-specific protein binding was blocked with 5 % non-fat dry milk for 1 h and then the membranes were incubated with anti-feline coronavirus nucleocapsid (N) antibody (FIPV3-70; MyBioSource, CA, USA) or anti-β-actin (Sigma-Aldrich) for 1 h. Antigen signals were visualized by reacting proteins on the membranes with horseradish peroxidase-conjugated anti-mouse IgG antibody (Promega) followed by an enhanced chemiluminescence substrate (SuperSignal West Femto Maximum Sensitivity Substrate; Thermo Scientific, Tokyo, Japan) according to the manufacturer’s protocol.

### Cell viability assay

We assayed WST-8 to evaluate cytotoxicity using the Cell Counting Kit-8 (Dojin Chemical Inc., Wako, Japan) according to the manufacturer’s directions.

### Statistical analysis

Statistical significance was determined using the Student’s *t* test. For all data analyzed, a significance threshold of *P* < 0.05 was assumed. The values are expressed in some figures as means ± standard deviation (SD).

## Results

### Inhibition of FIPV replication by CsA but not by FK506

**Figure 1:**
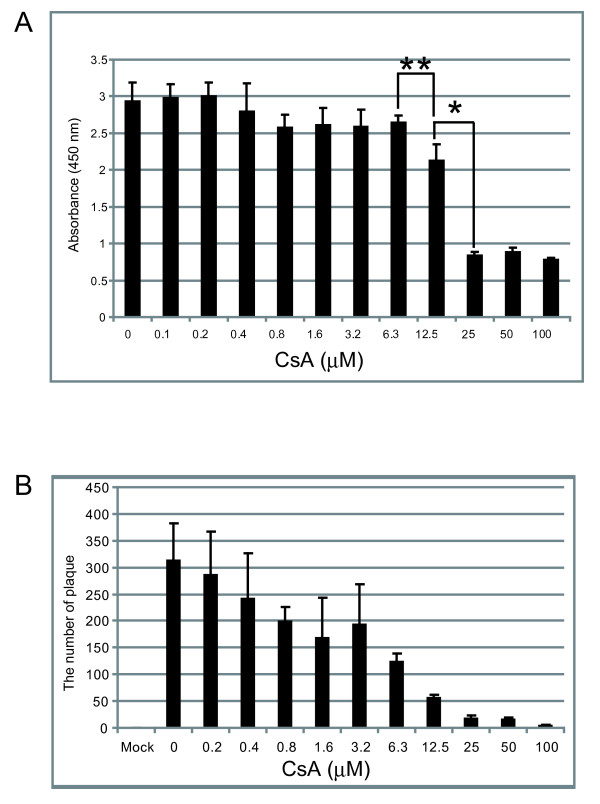
**Cyclosporin A inhibits cytopathic effects of FIPV on fcwf-4 cells.** (**A**) WST-8 assay of fcwf-4 cells cultured with indicated concentrations of CsA. Error bars indicate means ± 2SD. *P, ^**^P < 0.05. (**B**) Plaques counted in FIPV-infected fcwf-4 cells incubated with or without CsA. Cells infected with FIPV were incubated with various concentrations of CsA.

**Figure 2:**
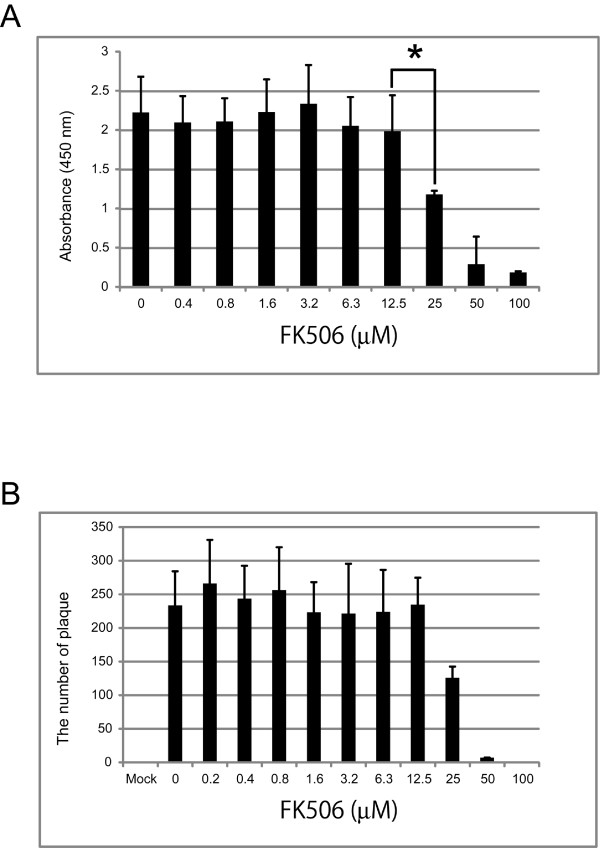
**FK506 does not inhibit cytopathic effects of FIPV on fcwf-4 cells.** (**A**) WST-8 assay of fcwf-4 cells cultured with various FK506 concentrations. Error bars indicate means ± 2SD. *P < 0.05. (**B**) The numbers of plaque were counted in FIPV-infected fcwf-4 cells incubated with or without FK506. Cells infected with FIPV were cultured with various concentrations of FK506.

We initially assessed the effects of CsA on FIPV RNA replication using cytotoxicity assays. Cyclosporin A at concentrations of 0–6.3 μM (cytotoxic dose_50_, 14.1 ± 1.72 μM) did not affect fcwf-4 cell viability (Figure 1A) and dose-dependently reduced the numbers of FIPV plaques (Figure 1B), whereas 10 μM CsA was slightly cytotoxic. Cyclosporin A blocks both the enzymatic activities of CyP that lead to the calcineurin (CN)-NF-AT and the P-glycoprotein pathway. We therefore assessed the effect of various concentrations of FK506, which also blocks the NF-AT pathway, on cell viability to confirm that CsA inhibited FIPV through this pathway. The cytopathic inhibitory effects did not significantly differ in the presence or absence of 0.08 - 10 μM FK506 (Figure 2B) and cell viability was not affected by 10 μM FK506 (Figure 2A).

**Figure 3:**
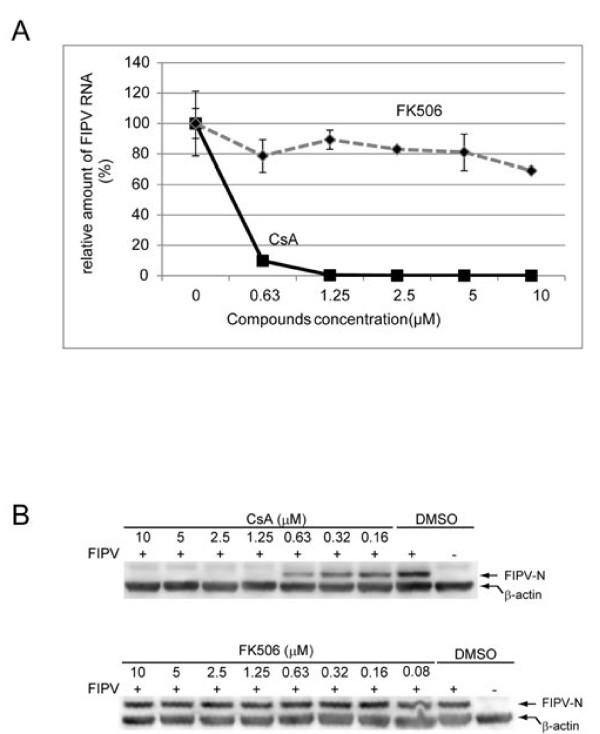
**Cyclosporin A, but not FK506 suppresses FIPV replication in fcwf-4 cells.** (**A**) Cells were infected with FIPV and incubated with or without the indicated concentrations of CsA or FK506. Total RNA extracted from cells 22 h later were reverse-transcribed to cDNA and assayed by real-time PCR assays targeting FIPV-N and β2M genes. Relative amounts of FIPV RNA expression data were normalized with β2M gene expression. Error bars indicate means ± 2SD. (**B**) Western blots of cells incubated with various concentrations of CsA and FK506. Lanes show total cellular proteins resolved by electrophoresis. Effects of CsA and FK506 were assessed using monoclonal anti-FIPV-N and anti-β-actin primary antibodies.

Quantitative RT-PCR showed that 0.63 - 10 μM CsA dose-dependently suppressed FIPV RNA replication, whereas FK506 did not exert significant inhibitory effects, except at 10 μM FK506 (approximately 30 % reduction compared to 0 μM FK506, *P* < 0.05; Figure 3A). Western blotting showed that CsA, but not FK506 dose-dependently decreased FIPV-N protein (Figure 3B).

We then examined whether the suppressive effects of CsA on FIPV replication depend on the inhibitory NF-AT pathway or P-glycoprotein pathway by incubating FIPV-infected cells with CsH, which specifically blocks the P-glycoprotein pathway. The results show that no inhibition occurred (data not shown).

### CsA does not elicit either an interferon-stimulated response or a NF-AT response in fcwf-4 cells

**Figure 4:**
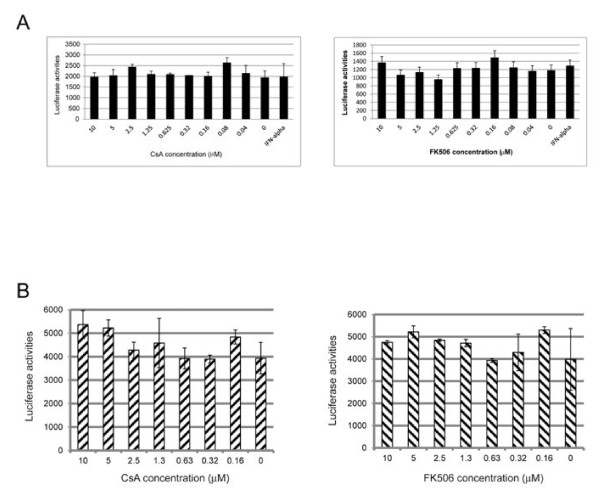
**CsA does not affect interferon-stimulated gene responses and NF-AT activities.** (**A**) Control vector pGL3 plasmid for normalization and ISRE-luciferase plasmid were transfected into fcwf-4 cells which were then incubated with or without indicated concentrations of CsA and FK506. Culture medium also contained interferon α (10 U/mL). Dual luciferase activities were measured at 48 h after transfection. (**B**) Plasmid vectors pGL4.30 [luc2P/NFAT-RE/Hygro] and pRL-SV40 for NF-AT response assays were transfected into fcwf-4 cells that were then cultured with or without indicated concentrations of CsA and FK506. Dual luciferase activities were measured at 48 h after transfection. Transfection efficiency was normalized by Renilla luciferase gene expression.

To determine whether the action of CsA and FK506 involves activation of interferon-stimulated gene responses in fcwf-4 cells, the ISRE-luciferase reporter plasmid, pISRE-TK hRluc (F) and pGL3 promoter plasmid as a normalization-control plasmid were transfected into fcwf-4 cells and cultured with feline interferon α, CsA or FK506. The results of the dual-luciferase assay showed that none of these factors significantly affected luciferase activities at 48 h after transfection. These results indicate that fcwf-4 cells are unresponsive, even to interferon α (Figure 4A). Consequently, the action of CsA on intracellular FIPV replication does not involve the activation of interferon-stimulated genes on fcwf-4 cells. Moreover, to evaluate the effects of CsA and FK506 on the calcineurin-NF-AT pathway in fcwf-4 cells, the NF-AT luciferase reporter plasmid, pGL4.30 [luc2P/NFAT-RE/Hygro] and pRL-SV40 as a normalization-control plasmid were transfected into fcwf-4 cells that had been incubated with CsA or FK506. Neither CsA nor FK506 affected NF-AT luciferase activities in fcwf-4 cells (Figure 4B). These findings show that CsA does not influence the NF-AT pathway in fcwf-4 cells and that the inhibition of FIPV RNA replication by CsA is independent of the calcineurin NF-AT pathway.

## Discussion

We discovered that CsA inhibits intracellular FIPV replication in vitro*.* The results of qRT-PCR and Western blotting showed that viral proliferation was strikingly inhibited at CsA concentrations between 0.16 and 10 μM. The results of cell viability assays showed that 10 μM CsA was slightly cytotoxic. The inhibitory effects at this concentration should therefore be considered in the light of cytotoxicity. In contrast, immunosuppressive FK506 did not inhibit FIPV replication except at 10 μM according to the qRT-PCR findings. However, the inhibitory effects of 0.08 - 10 μM FK506 did not significantly differ on Western blots and the number of plaques did not significantly differ within the same concentration range of FK506. These findings might be related to the difference of the analytical detection sensitivity between qRT-PCR and Western blot assays. Cyclosporin A binds cyclophilins, whereas FK506 binds cellular FKBP. Each complex independently inhibits the phosphatase activity of calcineurin that mediates the NF-AT pathway, which is critical to the expression of cytokines and their receptors [12,17]. The results of NF-AT reporter assays indicate that neither CsA nor FK506 influenced NF-AT activities on fcwf-4 cells under our experimental conditions. Thus, the antiviral activity against FIPV is not involved in the suppression of gene responses regulated by NF-AT but instead is exerted through distinct mechanisms that are independent of FK506. Pfefferle et al. recently described that SARS-coronavirus NSP1 overexpression increases signaling through the NF-AT pathway and enhances the induction of interleukin 2 [18]. Our data were somewhat inconsistent with these findings. The discrepancy might be explained in part by the fact that their experimental system included the addition of 13-O-Acetylphorbol 12-myristate (PMA) and ionomycin to the culture medium. Further knockdown studies of the NF-AT gene would clarify its role in FIPV proliferation. Furthermore, IFNα did not stimulate ISRE-promoter activities in fcwf-4 cells under these conditions. These data suggest that the action of CsA on the intracellular replication of FIPV is independent of the IFN pathway. This cell line might not be responsive to IFNα and would thus be useful for isolating feline coronavirus.

Cyclophilins have PPIase activity [13,19-21], contribute to the maturation of several proteins, and are involved in cell signaling, mitochondrial function (ATP synthesis), molecular chaperone activity, RNA splicing, stress response, gene expression, cholesterol transport, and the regulation of kinase activity [22-25]. Surface plasmon resonance technology and bioinformatics tools have found that the SARS coronavirus N protein binds to CyPA [26-28]. Therefore, the N protein of FIPV probably binds to CyP proteins to regulate viral replication. The roles of CyP in virus replication and the inhibitory effect by CsA on HCV and several other viruses have been studied [22-25]. The proposed mechanisms of the inhibitory effect of CsA mainly involve CypA and CypB in virus replication [23,29,30]. Based on these earlier findings, we believe that interaction between the FIPV genome is likely or that viral and CyP proteins play critical roles in viral replication and transcription. Further studies are required to resolve which CyP is critical for viral replication and which viral protein is required to form replication complexes with CyP and/or other cellular proteins.

Cats with clinically diagnosed FIP have very rarely been cured, although several therapeutic strategies have been attempted. Some cats treated with prednisolone and phenylalanine mustard or cyclophosphamide have gone into remission [26]. Various immunosuppressants, such as glucocorticoids and cyclophosphamide, may prolong life but do not alter the fatal outcome [4].

Further investigations using PPIase dominant-negative assays and RNA interference methods are warranted to clarify the role of CsA against PPIase in FIPV replication in vitro. In addition, whether CsA could be useful as FIP treatment in vivo remains to be determined

## Competing interests

The authors declare that they have no competing interests.

## Authors’ contributions

YT carried out all experiments, participated in the data collection and analysis, and prepared the manuscript. SO, MI, and ST carried out cell culture and virus preparation. YS performed some of the biochemical experiments and TS carried out luciferase assays. All authors read and approved the final manuscript.

## References

[B1] Rottier PJ Nakamura K Schellen P Volders H Haijema BJ Acquisition of macrophage tropism during the pathogenesis of feline infectious peritonitis is determined by mutations in the feline coronavirus spike protein J Virol 2005 79 14122 14130 10.1128/JVI.79.22.14122-14130.2005 16254347 PMC1280227

[B2] Pedersen NC Boyle JF Floyd K Fudge A Barker J An enteric coronavirus infection of cats and its relationship to feline infectious peritonitis Am J Vet Res 1981 42 368 377 6267960

[B3] Kennedy M Boedeker N Gibbs P Kania S Deletions in the 7a ORF of feline coronavirus associated with an epidemic of feline infectious peritonitis Vet Microbiol 2001 81 227 234 10.1016/S0378-1135(01)00354-6 11390106 PMC7117145

[B4] Pedersen NC A review of feline infectious peritonitis virus infection: 1963–2008 J Feline Med Surg 2009 11 225 258 10.1016/j.jfms.2008.09.008 19254859 PMC7129802

[B5] Berg AL Ekman K Belak S Berg M Cellular composition and interferon-gamma expression of the local inflammatory response in feline infectious peritonitis (FIP) Vet Microbiol 2005 111 15 23 10.1016/j.vetmic.2005.07.017 16183217 PMC7117157

[B6] Ritz S Egberink H Hartmann K Effect of feline interferon-omega on the survival time and quality of life of cats with feline infectious peritonitis J Vet Intern Med 2007 21 1193 1197 10.1111/j.1939-1676.2007.tb01937.x 18196725 PMC7197507

[B7] Hartmann K Ritz S Treatment of cats with feline infectious peritonitis Vet Immunol Immunopathol 2008 123 172 175 10.1016/j.vetimm.2008.01.026 18395801 PMC7132371

[B8] Billich A Fricker G Muller I Donatsch P Ettmayer P Gstach H Lehr P Peichl P Scholz D Rosenwirth B SDZ PRI 053, an orally bioavailable human immunodeficiency virus type 1 proteinase inhibitor containing the 2-aminobenzylstatine moiety Antimicrob Agents Chemother 1995 39 1406 1413 10.1128/AAC.39.7.1406 7492076 PMC162753

[B9] Billich A Hammerschmid F Peichl P Wenger R Zenke G Quesniaux V Rosenwirth B Mode of action of SDZ NIM 811, a nonimmunosuppressive cyclosporin A analog with activity against human immunodeficiency virus (HIV) type 1: interference with HIV protein-cyclophilin A interactions J Virol 1995 69 2451 2461 7884893 10.1128/jvi.69.4.2451-2461.1995 PMC188920

[B10] Bose S Mathur M Bates P Joshi N Banerjee AK Requirement for cyclophilin A for the replication of vesicular stomatitis virus New Jersey serotype J Gen Virol 2003 84 1687 1699 10.1099/vir.0.19074-0 12810862

[B11] Watashi K Ishii N Hijikata M Inoue D Murata T Miyanari Y Shimotohno K Cyclophilin B is a functional regulator of hepatitis C virus RNA polymerase Mol Cell 2005 19 111 122 10.1016/j.molcel.2005.05.014 15989969

[B12] Handschumacher RE Harding MW Rice J Drugge RJ Speicher DW Cyclophilin: a specific cytosolic binding protein for cyclosporin A Science 1984 226 544 547 10.1126/science.6238408 6238408

[B13] Zydowsky LD Etzkorn FA Chang HY Ferguson SB Stolz LA Ho SI Walsh CT Active site mutants of human cyclophilin A separate peptidyl-prolyl isomerase activity from cyclosporin A binding and calcineurin inhibition Protein Sci 1992 1 1092 1099 10.1002/pro.5560010903 1338979 PMC2142182

[B14] Rosenwirth B Oren DA Arnold E Kis ZL Eggers HJ SDZ 35–682, a new picornavirus capsid-binding agent with potent antiviral activity Antiviral Res 1995 26 65 82 10.1016/0166-3542(94)00066-H 7741522

[B15] Rosenwirth B Kis ZL Eggers HJ In vivo efficacy of SDZ 35–682, a new picornavirus capsid-binding agent Antiviral Res 1995 26 55 64 10.1016/0166-3542(94)00065-G 7741521

[B16] Ptak RG Gallay PA Jochmans D Halestrap AP Ruegg UT Pallansch LA Bobardt MD de Bethune MP Neyts J De Clercq E Dumont JM Scalfaro P Besseghir K Wenger RM Rosenwirth B Inhibition of human immunodeficiency virus type 1 replication in human cells by Debio-025, a novel cyclophilin binding agent Antimicrob Agents Chemother 2008 52 1302 1317 10.1128/AAC.01324-07 18212100 PMC2292519

[B17] Hait WN Harding MW Handschumacher RE Calmodulin, cyclophilin, and cyclosporin A Science 1986 233 987 989 10.1126/science.3016900 3016900

[B18] Pfefferle S Schopf J Kogl M Friedel CC Muller MA Carbajo-Lozoya J Stellberger T von Dall'Armi E Herzog P Kallies S Niemeyer D Ditt V Kuri T Züst R Pumpor K Hilgenfeld R Schwarz F Zimmer R Steffen I Weber F Thiel V Herrler G Thiel HJ Schwegmann-Wessels C Pöhlmann S Haas J Drosten C von Brunn A The SARS-coronavirus-host interactome: identification of cyclophilins as target for pan-coronavirus inhibitors PLoS Pathog 2011 7 1002331 10.1371/journal.ppat.1002331 PMC3203193 22046132

[B19] Lodish HF Kong N Cyclosporin A inhibits an initial step in folding of transferrin within the endoplasmic reticulum J Biol Chem 1991 266 14835 14838 1714445

[B20] Swanson SK Born T Zydowsky LD Cho H Chang HY Walsh CT Rusnak F Cyclosporin-mediated inhibition of bovine calcineurin by cyclophilins A and B Proc Natl Acad Sci U S A 1992 89 3741 3745 10.1073/pnas.89.9.3741 1315036 PMC525566

[B21] Zydowsky LD Ho SI Baker CH McIntyre K Walsh CT Overexpression, purification, and characterization of yeast cyclophilins A and B Protein Sci 1992 1 961 969 10.1002/pro.5560010801 1304384 PMC2142163

[B22] Fischer G Wittmann-Liebold B Lang K Kiefhaber T Schmid FX Cyclophilin and peptidyl-prolyl cis-trans isomerase are probably identical proteins Nature 1989 337 476 478 10.1038/337476a0 2492638

[B23] Gaither LA Borawski J Anderson LJ Balabanis KA Devay P Joberty G Rau C Schirle M Bouwmeester T Mickanin C Zhao S Vickers C Lee L Deng G Baryza J Fujimoto RA Lin K Compton T Wiedmann B Multiple cyclophilins involved in different cellular pathways mediate HCV replication Virology 2010 397 43 55 10.1016/j.virol.2009.10.043 19932913

[B24] Ivery MT Immunophilins: switched on protein binding domains? Med Res Rev 2000 20 452 484 10.1002/1098-1128(200011)20:6<452::AID-MED2>3.0.CO;2-6 11058892

[B25] Takahashi N Hayano T Suzuki M Peptidyl-prolyl cis-trans isomerase is the cyclosporin A-binding protein cyclophilin Nature 1989 337 473 475 10.1038/337473a0 2644542

[B26] Agresta BE Carter CA Cyclophilin A-induced alterations of human immunodeficiency virus type 1 CA protein in vitro J Virol 1997 71 6921 6927 9261419 10.1128/jvi.71.9.6921-6927.1997 PMC191975

[B27] Braaten D Franke EK Luban J Cyclophilin A is required for an early step in the life cycle of human immunodeficiency virus type 1 before the initiation of reverse transcription J Virol 1996 70 3551 3560 8648689 10.1128/jvi.70.6.3551-3560.1996 PMC190230

[B28] Streblow DN Kitabwalla M Malkovsky M Pauza CD Cyclophilin a modulates processing of human immunodeficiency virus type 1 p55Gag: mechanism for antiviral effects of cyclosporin A Virology 1998 245 197 202 10.1006/viro.1998.9155 9636359

[B29] De BP Thornton GB Luk D Banerjee AK Purified matrix protein of vesicular stomatitis virus blocks viral transcription in vitro Proc Natl Acad Sci U S A 1982 79 7137 7141 10.1073/pnas.79.23.7137 6296818 PMC347293

[B30] Luo C Luo H Zheng S Gui C Yue L Yu C Sun T He P Chen J Shen J Luo X Li Y Liu H Bai D Shen J Yang Y Li F Zuo J Hilgenfeld R Pei G Chen K Shen X Jiang H Nucleocapsid protein of SARS coronavirus tightly binds to human cyclophilin A Biochem Biophys Res Commun 2004 321 557 565 10.1016/j.bbrc.2004.07.003 15358143 PMC7092810

